# Prognostic impact of tumor size on isolated hepatocellular carcinoma without vascular invasion may have age variance

**DOI:** 10.3389/fsurg.2022.988484

**Published:** 2023-01-06

**Authors:** Yi Zhang, Jun-Gang Zhang, Wei Yu, Lei Liang, Chun Wu, Cheng-Wu Zhang, Ya-Ming Xie, Dong-Sheng Huang, Ying Shi

**Affiliations:** ^1^ Zhejiang Provincial People's Hospital, Qingdao University, Hangzhou, China; ^2^ General Surgery, Cancer Center, Department of Hepatobiliary & Pancreatic Surgery and Minimally Invasive Surgery, Zhejiang Provincial People's Hospital, Affiliated People's Hospital, Hangzhou Medical College, Hangzhou, China; ^3^ Key Laboratory of Gastroenterology of Zhejiang Province, Zhejiang Provincial People's Hospital, Affiliated People's Hospital, Hangzhou Medical College, Hangzhou, China; ^4^ Obstetrics and Gynecology, Zhejiang Provincial People's Hospital, Affiliated People's Hospital, Hangzhou Medical College, Hangzhou, China

**Keywords:** hepatocellular carcinoma, tumor size, *P* for interaction, *P* for trend, per 1 SD

## Abstract

**Background:**

Previous studies suggested that tumor size was an independent risk factor of prognosis for hepatocellular carcinoma (HCC). However, the general prognostic analysis did not consider the interaction between variables. The purpose of this study was to investigate whether the effect of tumor size on the prognosis of isolated HCC without vascular invasion varies according to covariates.

**Methods:**

Patients were selected from the Surveillance, Epidemiology, and End Results (SEER) database to investigate whether there was an interaction between age and tumor size on the prognosis. Then the trend test and the value of per 1 SD of tumor size were calculated. In addition, the data of Zhejiang Provincial People's Hospital meeting the requirements were selected to verify the obtained conclusions.

**Results:**

Multivariable Cox regression analysis of the database cohort showed that age, gender, tumor size, pathological grade and marital status were independent risk factors for prognosis. Interaction test showed that there was an interaction between age and tumor size (*P* for interaction < 0.05). Stratified analysis by age showed that tumor size was an independent risk factor for prognosis when age ≤65 years old (HR:1.010,95%CI1.007–1.013 *P* < 0.001), while tumor size was not an independent risk factor for prognosis when age >65 years old. This result was confirmed by trend analysis (*P* for trend < 0.001), and the prognostic risk increased by 42.1% for each standard deviation increase of tumor size among patients age ≤65 years. Consistent conclusion was obtained by multivariable cox regression analysis and interaction test on the verification cohort. In the validation cohort, for each standard deviation increase of tumor size in patients ≤65 years old, the risk of prognosis increased by 52.4%.

**Conclusion:**

Tumor size is not an independent risk factor for the prognosis of isolated HCC without vascular invasion when patient's age >65 years. Therefore, when analyzing the relationship between tumor size and prognosis, stratified analysis should be performed according to age.

## Introduction

The incidence of hepatocellular carcinoma (HCC) is increasing year by year and has become the second leading cause of cancer death worldwide (1, 2). Globally, about 900,000 new cases and 830,000 deaths occur each year (3). What is more, according to Surveillance, Epidemiology, and End Results (SEER) database, the annual incidence of HCC increased by 3.1% per year from 2008 to 2012, with 11.5 and 3.9 HCC diagnoses per 100,000 males and females respectively, and the death rates increased 2.8% and 3.4% per year in males and females respectively, and increased at the highest rate of all cancers during this time (4). Globally, the incidence and death rates of HCC are different between males and females. Males are more likely to develop HCC than females, especially in some regions with a high incidence of liver cancer, reaching a 3.7 to 1 ratio (5). In addition, patients with underlying liver disease are more likely to develop HCC due to hepatitis B or C virus (HBV or HCV) infection (6).

With the advancement of medical technology and in-depth research on HCC, doctors have more options to choose from when treating this disease. Radical resection of the tumor is still the most commonly used method, other treatments include transarterial chemoembolization, radiofrequency ablation, microwave ablation, percutaneous ethanol injection, systemic chemotherapy, radiotherapy, and molecular targeted therapy (7). Tumor size is always considered in the selection and application of these therapies. Tumor size largely determines diagnosis, treatment, and prognosis, so many staging systems take tumor size into account, such as the Barcelona Clinic Liver Cancer staging classification (BCLC) (8), the Okuda staging system (9), the Cancer of the Liver Italian Program (CLIP) (10), the Japan Integrated Staging Score (JIS) (11), and the American Joint Committee on Cancer (AJCC) staging system (12). Thus, tumor size is very important in clinical practice.

Tumor size is a continuous variable, which is often translated directly into categorical variables in these staging systems. In the eighth edition of the AJCC staging system, tumor size was translated into categorical variables with 2 cm and 5 cm as cutoff values. This coarse classification may obscure a nuanced description of the effects of tumor size across the full range of possible sizes. Therefore, more accurate treatment of tumor size is needed when analyzed the relationship between tumor size and prognosis (13, 14). In another study, tumor size was divided into more detailed groups with cutoff values of 10, 20, 30, and 40 mm (15). This grouping method of continuous variables can better analyze the data, but there was no trend analysis of tumor size in this study. In our study, we converted tumor size into ordinal categorical variables in terms of quartiles and conducted trend tests, and then standardized tumor size for multivariable analysis.

Tumor size can not only directly affect the prognosis of patients, but also may affect other variables. One study showed that adjuvant TACE improved the prognosis of HCC patients with microvascular invasion, but not when the tumor size was >5cm (16). In addition, literatures have shown a significant positive correlation between tumor size and distant metastasis, especially that tumor size ≥58 mm is an independent risk predictor of distant metastasis of HCC, and the prognosis of patients will deteriorate with the increase of tumor size (17). To eliminate the interaction between variables, we tested the interaction between tumor size and other variables. If the values of *P* for interaction >0.05, it can be considered that there is no interaction between this variable and tumor size, that is, the results of stratified studies cannot be considered to be different. On the contrast, when the values of *P* for interaction <0.05, it is considered that there is an interaction between this variable and tumor size, that is, there are differences in the results of studies at different levels. At this point, it can be considered that this variable is an important confounding factor affecting the relationship between tumor size and prognosis of patients, and it is necessary to conduct stratified analysis of patients to obtain more accurate results.

## Materials and methods

### Participants

Data Source were extracted from SEER database(http://seer.cancer.gov/). SEER-Stat [version 8.3.8 (National Cancer Institute, Bethesda, Maryland, United States)] was used to filter and collect the information of the patients. Patients selected were diagnosed with HCC from 2010 to 2015 who underwent surgical resection and had complete follow-up. Subjects in this study were identified in the SEER database as patients with “(Site and Morphology. Site recode ICD-O-3/WHO = 2008) = Liver, Stage. TNM. Derived AJCC T 7th ed. (2010+)= T1 and so on”. The AJCC Cancer Staging has been updated to the 8th edition at present (18), while T1 of 7th Edition is defined as an isolated tumor without vascular invasion, which also meets our screening criteria. When selecting outcome variables, our screening condition was “COD to site recode = Liver”. This means that if the patient had a death outcome, it was caused by HCC rather than other causes, which can better control confounding factors. When we got the original data, the data were sorted out and patients with survival time of 0 months and unknown values (such as tumor grade and tumor size, etc.) were deleted. After screening, there were 1,920 patients who met our requirements. The validation cohort selected HCC patients from Zhejiang Provincial people's Hospital from 2010 to 2015. Finally, 707 patients met the requirements.

### Study design and outcomes

Univariate and multivariable analyses were performed on patients obtained from SEER database to identify independent risk factors for prognosis. We used the method of visual binning to transform tumor size and age into quartiles and quintiles. The whole cohort was stratified for each variable and a multivariable adjusted trend test was performed at each level to determine whether tumor size was an independent risk factor for patient outcome. In order to judge whether the stratified analysis is reasonable, that is, whether the HR value difference between each stratification is statistically significant, the value of *P* for interaction is calculated. If there was no interaction between variables and tumor size, the effect of tumor size on patient outcomes was consistent in each subgroup, that is, we did not need to stratify patients in our analysis. On the contrast, if there was an interaction between variables and tumor size, it indicated that the effect of tumor size on patient prognosis was inconsistent in different subgroups, and patients should be stratified according to variables.

After the interaction test, we found an interaction between age and tumor size. Therefore, we divided the whole cohort into two cohorts according to age, and conducted univariate and multivariable analyses respectively. In the subgroup where tumor size was an independent risk factor for patient prognosis, trend analysis of tumor size was performed again and the value of per 1 SD was calculated after tumor size was standardized. In univariate and multivariable analyses of subgroups, patient age was analyzed as a continuous variable within each subgroup. We used the same method to perform univariate and multivariable cox regression on the data of the verification cohort to obtain the risk factors for the prognosis of the patients. The interaction test between tumor size and age was performed to verify whether there was an interaction between tumor size and age. Then the validation cohort was stratified according to age to prove that there was an age difference between tumor size and prognosis. Finally, the trend test and the per 1 SD value of the validation cohort were calculated.

### Statistical analysis

We did some transformation of the variables. Age was a continuous variable, and the median age of patients in the database cohort was 62 years. In most studies, patients older than 65 years were considered elderly, so we also converted age to a dichotomous variable with 65 years as a cut-off, combined with the median age in the database cohort. Furthermore, we converted race into a binary variable (white, others), and marital status into a binary variable (currently married, currently single for whatever reasons). We describe the basic characteristics of the whole cohort, with continuous variable data expressed as median (quartile range) and categorical variable data expressed as frequency (percentage). In addition, in the validation cohort, the optimal cut-off value for intraoperative blood loss was found to be 200 ml using the ROC curve.

In the study of the cohort, univariate and multivariable analyses were performed by Cox regression analysis. In order to prevent meaningful indicators from being excluded in the univariate analysis, we considered that *P* ≤ 0.15 was statistically significance. Multivariable analyses were performed by Cox regression analysis with a forward: LR and *P* ≤ 0.05 was considered statistically significance. In trend analysis, we gradually adjusted the model by multiple factors through model 1 and model 2, and finally obtained the value of *P* for trend, HR and 95% CI. To calculate per 1 SD, tumor size in the study cohort was standardized and then adjusted for HR and 95% CI by multivariable analysis.

All *P* values were two-tailed. The following software was used for the analysis: IBM SPSS version 26.0 (IBM, United States) and R version 4.1.2.

## Results

### Baseline characteristics

**Figure 1:**
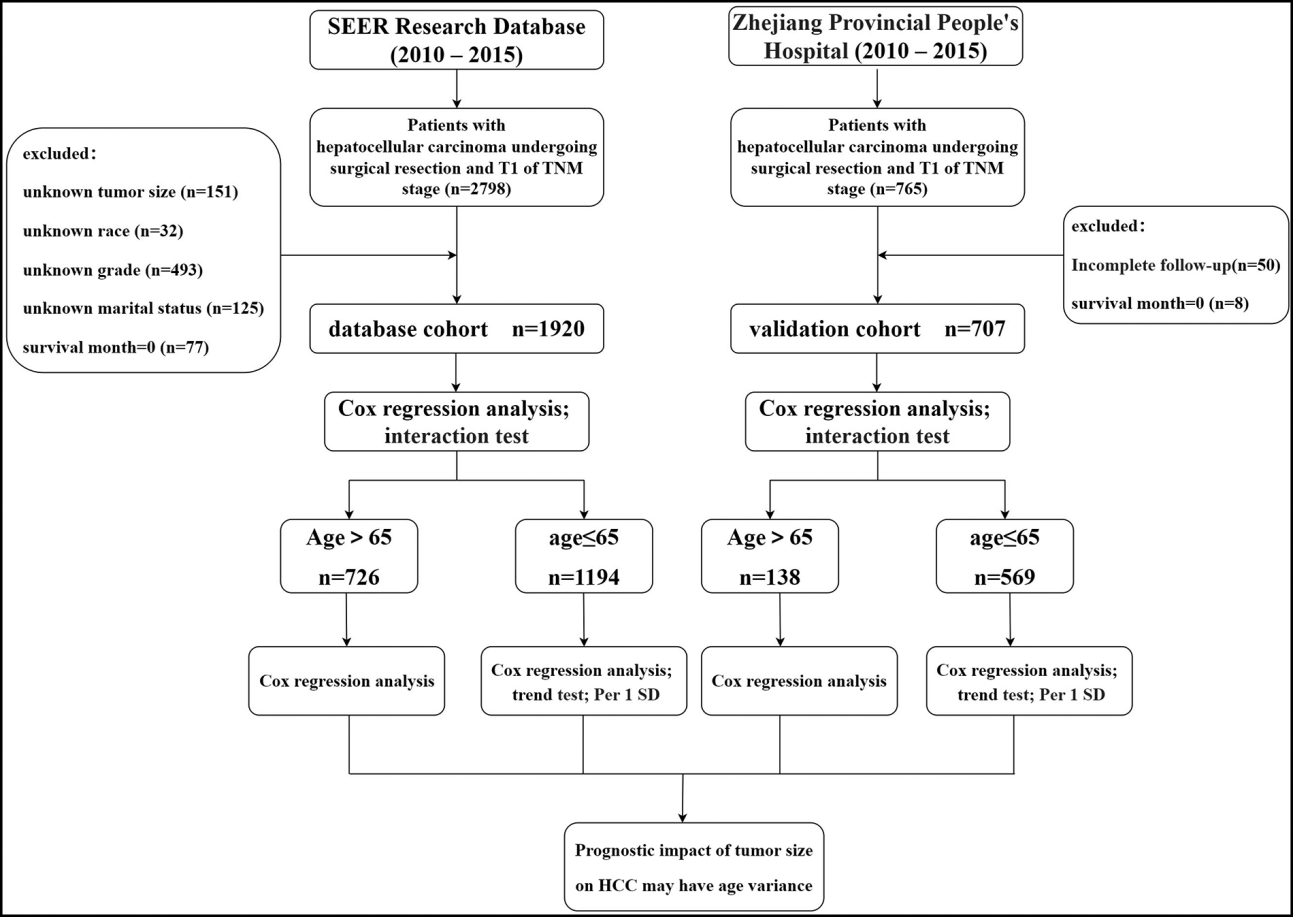
Flow chart of patient inclusion and main analysis.

We identified 1,920 eligible HCC patients in SEER database during a 6-year study period (between 2010 and 2015). Patients with missing information on tumor size, race, pathological grade, and marital status were deleted through screening. In addition, we excluded patients who died during postoperative hospitalization (survival month = 0). Finally, we ended up with 1,920 patients. In the validation cohort, 707 eligible patients were finally obtained after excluding the patients who did not meet the screening criteria. The filtering process and the main operation flow are shown in Figure 1.

In the SEER database cohort, the median age of the patients was 62 years, the median tumor size was 35 mm, 1,218 were white (63.4%), 1,383 were male (72.0%), and 1,233 were married (64.2%). In addition, the number of patients at G2 stage was the highest (1,037, 54.0%), and that at G4 stage was the lowest (22, 1.1%). In the validation cohort, the median age of patients was 57 years, the median tumor size was 35 mm, 604 were men (85.4%), 376 (53.2%) patients with internal blood loss >200 ml, and 467 (66.1%) patients with PA-TACE. In addition, the number of patients in G2 was the largest, with 436 (61.7%), and that in G4 was the smallest, with only 16 (2.3%).The main results are shown in Table 1.

**Table 1:** Clinicopathological characteristics of SEER database cohort and validation cohort.

Variables	SEER (*n* = 1,920)	Validation (*n* = 707)
Age, years^a^	62 (56–69)	57 (51–64)
White, *n* (%)	1,218 (63.4)	–
Males, *n* (%)	1,383 (72.0)	604 (85.4)
Tumor size, mm^a^	35 (24–55)	35 (22–60)
Grade, *n* (%)		
1	575 (29.9)	79 (11.2)
2	1,037 (54.0)	436 (61.7)
3	286 (14.9)	176 (24.9)
4	22 (1.1)	16 (2.3)
Married, *n* (%)	1,233 (64.2)	–
Intraoperative blood loss >200 ml	–	376 (53.2)
Post-operation TACE	–	467 (66.1)

^a^ Median and interquartile range.

### Multivariable analysis and interaction test

Multivariable Cox regression analysis was performed on the data of the database, and the following five factors were found to be independent prognostic factors for HCC (Table 2): age (HR, 1.343; 95% CI, 1.028,1.753), gender (HR, 1.429; 95% CI, 1.040,1.965), pathological grading (G2: HR, 1.127; 95% CI, 0.806–1.575. G3: 2.729; 95% CI, 1.881–3.959. G4: HR, 3.937; 95% CI, 1.849–8.358), tumor size (HR, 1.009; 95% CI, 1.006,1.011), and marital status(HR, 0.698; 95% CI, 0.533,0.914). However, no statistical difference was observed with regard to race (univariate analysis *P* = 0.388). Multivariable Cox regression analysis was performed on the data of the validation cohort, and the following 5 factors were found to be independent prognostic factors for HCC (Supplementary Table S1): age (HR, 1.030; 95% CI, 1.016–1.045), tumor size (HR, 1.009; 95% CI, 1.006–1.013), pathological grading (G2: HR, 2.630; 95% CI, 1.418–4.877, G3: 2.912; 95% CI, 1.511–5.612, G4: HR, 1.169; 95% CI, 0.352–3.888), intraoperative blood loss (HR, 1.617; 95% CI, 1.169–2.236), and PA-TACE (HR, 0.721; 95% CI, 0.538–9.965).

**Table 2:** Univariate and multivariable analyses of SEER database cohort.

Variables	Univariate analysis		Multivariable analysis	
	HR (95% CI)	*P* value	HR (95% CI)	*P* value
Age	1.520 (1.170–1.975)	**0** **.** **002**	1.343 (1.028–1.753)	**0**.**030**
Male	1.277 (0.933–1.748)	**0**.**127**	1.429 (1.040–1.965)	**0**.**028**
White	1.126 (0.860–1.475)	0.388	** **	** **
Tumor size	1.009 (1.007–1.012)	**<0**.**001**	1.009 (1.006–1.011)	**<0**.**001**
Grade				
1	1.000	** **	1.000	
2	1.230 (0.881–1.716)	**0**.**224**	1.127 (0.806–1.575)	**0**.**485**
3	2.955 (2.043–4.275)	**<0**.**001**	2.729 (1.881–3.959)	**<0**.**001**
4	5.594 (2.656–11.783)	**<0**.**001**	3.937 (1.849–8.358)	**<0**.**001**
Married	0.765 (0.587–0.996)	**0**.**047**	0.698 (0.533–0.914)	**0**.**009**

Tumor size was quartile grouped by using the method of visual. The quartiles of database cohort were Q1 (≤24 mm), Q2 (25–35 mm), Q3 (36–55 mm), Q4 (≥56 mm). The quartiles of the validation cohort were Q1 (≤22 mm), Q2 (23–35 mm), Q3 (36–60 mm), Q4 (≥61 mm). In stratified analysis, tumor size had a trend in patients ≤ 65 years (*P* for trend <0.05), while it did not have a trend in patients >65 years old (Table 3). Similarly, in the validation cohort, tumor size had a trend in patients ≤65 years old, while it had no trend in patients >65 years old (Supplementary Table S2). Interaction analysis found that there was interaction between age and tumor size in both cohorts (*P* for interaction <0.001).

**Table 3:** Test for trend and interaction between tumor size and other variables in SEER database cohort.

Variables	Tumor size				*P* for trend	*P* for interaction
	Q1	Q2	Q3	Q4		
	(≤24 mm)	(25–35 mm)	(36–55 mm)	(≥56 mm)		
**Age**					** **	**0**.**001**
≤65	1.000	1.457 (0.779–2.725)	2.706 (1.499–4.886)	4.951 (2.888–8.488)	**<0.001**	** **
>65	1.000	1.036 (0.518–2.074)	0.921 (0.462–1.836)	1.441 (0.755–2.751)	0.180	
**Grade**						0.179
G1	1.000	0.984 (0.429–2.255)	1.079 (0.476–2.445)	1.579 (0.742–3.361)	**0.027**	** **
G2	1.000	1.468 (0.743–2.900)	1.898 (0.977–3.687)	3.772 (2.058–6.911)	**<0.001**	** **
G3	1.000	2.907 (0.826–10.231)	4.809 (1.404–16.466)	7.851 (2.380–25.900)	**<0.001**	** **
G4	–	–	–	–	0.932	
**Married**						0.841
Married	1.000	1.315 (0.706–2.447)	2.017 (1.107–3.676)	3.304 (1.896–5.757)	**<0.001**	** **
Others	1.000	1.685 (0.838–3.387)	1.924 (0.966–3.832)	3.515 (1.859–6.644)	**<0.001**	
**Sex**					** **	0.394
Female	1.000	0.939 (0.313–2.816)	2.139 (0.812–5.637)	3.587 (1.442–8.922)	**<0.001**	** **
Male	1.000	1.630 (0.976–2.721)	1.882 (1.127–3.143)	3.410 (2.133–5.452)	**<0.001**	** **
**Race**					** **	0.890
White	1.000	1.889 (1.047–3.409)	2.168 (1.190–3.949)	3.704 (2.124–6.458)	**<0.001**	** **
Others	1.000	0.949 (0.436–2.065)	1.631 (0.806–3.299)	3.016 (1.572–5.786)	**<0.001**	** **

### Relationship between tumor size and CSS at different stratified of age

Multivariable Cox regression analysis was performed for patients aged ≤65 years in the database cohort, and the following 3 factors were found to be independent prognostic factors for HCC (Table 4): tumor size (HR, 1.010; 95% CI, 1.007–1.013), pathological grading (G2: HR, 1.472; 95% CI, 0.922–2.350, G3: 4.063; 95% CI, 2.448–6.746, G4: HR, 8.175; 95% CI, 3.076–21.722), and marital status(married: HR, 0.671; 95% CI, 0.469–0.959). Multivariable Cox regression analysis was performed for patients aged ≤65 years in the validation cohort, and the following 4 factors were found to be independent prognostic factors for HCC (Supplementary Table S3): tumor size (HR, 1.010; 95% CI, 1.007–1.014), pathological grading (G2: HR, 2.880; 95% CI, 1.264–6.566, G3: 2.761; 95% CI, 1.145–6.660, G4: HR, 6.456; 95% CI, 1.758–23.707), intraoperative blood loss(HR, 1.760; 95% CI, 1.180–2.625) and PA-TACE(HR, 0.662; 95% CI, 0.470–0.993).

**Table 4:** Univariate and multivariable analyses of the two subgroups with different ages in SEER database cohort.

Variables	Univariate analysis		Multivariable analysis	
	HR (95% CI)	*P* value	HR (95% CI)	*P* value
**Age ≤65**				
Male	1.343 (0.860–2.097)	0.195		
White	1.429 (0.999–2.045)	**0**.**051**	** **	** **
Tumor size	1.010 (1.007–1.013)	**<0**.**001**	1.010 (1.007–1.013)	**<0**.**001**
Grade				** **
1	1.000	** **	1.000	** **
2	1.592 (1.000–2.437)	**0**.**050**	1.472 (0.922–2.350)	**0**.**105**
3	4.329 (2.611–7.179)	**<0**.**001**	4.063 (2.448–6.746)	**<0**.**001**
4	12.105 (4.640–31.580)	**<0**.**001**	8.175 (3.076–21.722)	**<0**.**001**
Married	0.679 (0.477–0.968)	**0**.**032**	0.671 (0.469–0.959)	**0**.**029**
**Age >65**				** **
Male	1.280 (0.821–1.998)	0.276		** **
White	0.824 (0.543–1.251)	0.364		** **
Tumor size	1.007 (1.002–1.011)	0.706		** **
Grade				** **
1	1.000	**0**.**020**		** **
2	0.764 (0.474–1.232)	**0**.**270**		** **
3	1.561 (0.906–2.689)	**0**.**109**		** **
4	1.903 (0.576–6.290)	**0**.**292**		** **
Married	0.843 (0.564–1.261)	0.407		

Model 1: Adjusted for age.

Model 2: Adjusted for age, race, grade and marital status.

Trend test was conducted for all patients in the database and patients with age ≤65 respectively, and it was found that tumor size was an independent risk factor for prognosis, and both had trend (Table 5). After standardized tumor size, the multivariable analysis showed that the prognosis risk of patients age ≤65 and the total cohort increased by 42.1% and 39.2% respectively for per 1 SD increase. The trend test was carried out for all patients in the validation cohort and patients with age ≤65. It was also found that tumor size was an independent risk factor for prognosis and had a trend (STab4). After standardized tumor size, multivariable analysis showed that the prognosis risk of patients in the cohort with age ≤65 and the total cohort increased by 52.4% and 40.4% respectively for every increase of per 1SD.

**Table 5:** Trend test and the values of per 1 SD of the whole cohort and the subgroup in SEER database cohort.

Tumor size	*n*	HR (95% CI)	
		Model 1	Model 2
**Whole cohort**	1,920		
Q1 (≤24 mm)	509	1.000	1.000
Q2 (25–35 mm)	483	1.588 (1.001–2.522)	1.475 (0.982–2.344)
Q3 (36–55 mm)	464	2.137 (1.362–3.352)	1.983 (1.263–3.113)
Q4 (≥56 mm)	464	3.765 (2.482–5.684)	3.412 (2.248–5.179)
*p* for trend		**<0.001**	**<0.001**
Per 1 SD		1.404 (1.285–1.534)	1.392 (1.271–1.524)
**Age ≤65**	1,194		
Q1 (≤21 mm)	320	1.000	1.000
Q2 (22–31 mm)	278	1.590 (0.778–3.249)	1.327 (0.646–2.724)
Q3 (32–49 mm)	298	2.800 (1.460–5.369)	2.266 (1.175–4.373)
Q4 (≥50 mm)	298	6.022 (3.307–10.966)	4.818 (2.627–8.835)
*P* for trend		**<0.001**	**<0.001**
Per 1 SD		1.445 (1.291–1.616)	1.421 (1.261–1.601)

Model 1: Adjusted for age, sex.

Model 2: Adjusted for age, sex, race, grade and marital status.

## Discussion

Based on the analysis of the database cohort and validation cohort, we found that the effect of tumor size on the prognosis of patients with isolated HCC without vascular invasion have age variance. Tumor size is a prognostic risk factor for patients aged <65 years, but not for patients aged ≥65 years.

Tumor size is an important clinical parameter, which will be firstly taken into account in the formulation of tumor stage, the selection of treatment options, or the construction of prediction models. Studies on different types of tumors have shown that tumor size is an independent prognostic risk factor (19–22). Tumor size is a relatively easy obtained clinical indicator, which can be accurately measured by imaging before surgery (23). It is important to note that size is not the only morphological feature about tumors. Whether the tumor is isolated, whether there is vascular invasion, lymph node metastasis, distant metastasis, etc., must be considered. If we ignore the diversity of tumor features and focus only on the impact of tumor size on the prognosis, the results will be biased by confounders. In order to avoid the influence of these confounders, we only studied isolated HCC without vascular invasion in this study, which could eliminate the interference of excessive confounders and make the results of our analysis more accurate.

With the advance of imaging technology, it is possible to assess whether the tumor is isolated, vascular invasion, lymph node metastasis, distant metastasis and other conditions before surgery (24). This can provide a reference for doctors to formulate more personalized treatment plans, and also enable patients to fully understand their conditions, and cooperate with doctors to make the most reasonable decisions so as to achieve the most satisfactory results. Many studies have shown that tumor size is an independent risk factor for prognosis of HCC (25–28). However, these studies analyzed tumor size as continuous or binary variable. When tumor size was analyzed as continuous variable, the HR values represented the increased risk of prognosis for each 1 mm increase with tumor size. The changes may be too minor to be of practical clinical significance. When the continuous variable is converted into binary variable, the improper cut-off value may affect the result. After comprehensive consideration, we adopted a variety of variable conversion methods to analyze tumor size in our study (29). First of all, tumor size was converted to ordinal categorical, and then trend analysis and trend test were performed. Then, per 1 SD was calculated after tumor size was standardized. Finally, in the stratified analysis, tumor size was used as a continuous variable for multivariable analysis. In this way, tumor size can be verified multiple times as a variable of different types, so as to prevent the error of results caused by the change of variable types (30).

When we conduct prognostic analysis, confounding factors need to be controlled. The Cox regression analysis is a common method to control confounding factors. However, it is impossible to judge whether there exists interaction between variables through univariate and multivariable analysis. Therefore, we conducted analyze of interaction. When *P* for interaction >0.05, it indicated that there was no interaction between variables, that was, there was no difference in the results of stratified analysis or overall analysis of patients. When *P* for interaction <0.05, it indicated that there existed interaction between variables, that was, this variable was an important confounding factor and had an important influence on the results of our analysis (31).

Elderly patients are often accompanied by frailty, with significantly poorer nutritional status and postoperative recovery than younger patients (32, 33). In addition, the nutritional absorption of elderly patients after surgery was worse than that of younger patients (34). These adverse factors will delay postoperative recovery and early ambulation time in elderly patients, and slow recovery and delayed ambulation will worsen the prognosis of patients. More importantly, elderly patients tend to have underlying diseases (35). These chronic underlying diseases coexist with the patient for a long time and maintain a state of balance, but when stress occurs, such as a major trauma such as surgery, this balance will be upset and the patient's physical state will decline rapidly. This may cause that the effect of tumor size on the prognosis of patients has not been shown, and patients show a poor prognosis due to the decline of their own physical fitness. This may explain why tumor size is not an independent risk factor in elderly.

The clinical value of this study is that it can more accurately analyze the prognostic risk factors of isolated HCC without vascular invasion. With the improvement of imaging technology, preoperative examination can determine the tumor size, whether it is isolated or not, and whether there is vascular invasion (36). Tumor size should be considered as an important indicator in the treatment and prognosis prediction of patients aged ≤65 years with isolated HCC without vascular invasion. However, tumor size should not be considered as an important predictor for prognosis aged >65 years with isolated HCC without vascular invasion. In this study, we were unable to identify independent risk factors for prognosis in elderly patients. We will explore the prognostic risk factors in elderly patients in the following study. Therefore, stratification of patients according to age, whether prevention or treatment, can provide more benefit to patients.

## Conclusion

Through the analysis of the database cohort and verification cohort, we found that there may be age differences in the effect of tumor size on the prognosis of isolated HCC without vascular invasion. In stratified analysis by age of 65 years, tumor size was an independent risk factor for prognosis in the cohort aged ≤65 years, but was not an independent risk factor for prognosis when age > 65 years. Therefore, stratified analysis should be performed to analyze the relationship between tumor size and CSS of isolated HCC without vascular invasion. Proper stratification analysis can control the interference of confounding factors to a large extent and improve the accuracy of prediction model.

## Limitation

The main subjects in this study were isolated HCC without vascular invasion, and we did not analyze all kinds of HCC. There may be more complex interactions between tumor size and other variables when there is multiple tumors or vascular invasion. In addition, we only analyzed part of the variables and failed to collect all the variables that might affect the prognosis of patients, so there may be other variables interacting with tumor size. In addition, we were not able to identify prognostic risk factors in older patients, which will be continued in further studies.

## Data Availability

The datasets presented in this study can be found in online repositories. The names of the repository/repositories and accession number(s) can be found in the article/**Supplementary Material**.
