# Supplementary material for: Prognostic impact of tumor size on isolated hepatocellular carcinoma without vascular invasion may have age variance
Source: Front Surg. 2023 Jan 6;9:988484. doi: 10.3389/fsurg.2022.988484 (PMC9852506; doi:10.3389/fsurg.2022.988484)
Supplement: Supplementary file 1 [file Table1.docx]

Supplementary table 1. Univariate and multivariable analyses of validation cohort.

| Variables | Univariate analysis | | | | Multivariable analysis | |
| --- | --- | --- | --- | --- | --- | --- |
|  | HR (95% CI) | | | P value | HR (95% CI) | P value |
| Age | 1.030(1.015-1.045) | | | **<0.001** | 1.030(1.016-1.045) | **<0.001** |
| Tumor size | 1.010(1.007-1.013) | | | **<0.001** | 1.009(1.006-1.013) | **<0.001** |
| Grade |  | | |  |  |  |
| 1 | Ref | | |  | Ref |  |
| 2 | 2.410(1.303-4.457) | | | **0.005** | 2.630(1.418-4.877) | **0.002** |
| 3 | 2.921(1.519-5.618) | | | **0.001** | 2.912(1.511-5.612) | **0.001** |
| 4 | 2.852(0.907-8.971) | | | **0.073** | 1.169(0.352-3.888) | **0.799** |
| Intraoperative blood loss | | |  |  |  |  |
| ≤200ml | Ref | | |  | Ref |  |
| >200ml | 1.965(1.442-2.676) | | | **<0.001** | 1.617(1.169-2.236) | **0.004** |
| PA-TACE | |  | |  |  |  |
| No | Ref | | |  | Ref |  |
| Yes | 0.768(0.578-1.020) | | | **0.068** | 0.721(0.538-9.965) | **0.028** |

PA-TACE :postoperative adjuvant transcatheter arterial chemoembolization

Supplementary table 2. Test for trend and interaction between tumor size and age in validation cohort.

| Variables | Tumor size | | | | P for trend | P for interaction |
| --- | --- | --- | --- | --- | --- | --- |
|  | Q 1 Q 2 Q 3 Q 4  (≤22 mm) (23-35 mm) (36-60 mm) (≥61 mm) | | | |  |  |
| **Age** |  |  |  |  |  | **0.017** |
| ≤65 | 1.000 | 1.027(0.585-1.804) | 1.878(1.108-3.184) | 3.219(1.949-5.317) | **< 0.001** |  |
| >65 | 1.000 | 0.913(0.313-2.665) | 1.085(0.415-2.837) | 0.923(0.309-2.753) | 0.969 |  |

Supplementary table 3. Univariate and multivariable analyses of the two subgroups with different ages in validation cohort.

| Variables | Univariate analysis | | | | Multivariable analysis | |
| --- | --- | --- | --- | --- | --- | --- |
|  | HR (95% CI) | | | P value | HR (95% CI) | P value |
| **Age≤65** |  | | |  |  |  |
| Tumor size | 1.102(1.009-1.016) | | | **< 0.001** | 1.010(1.007-1.014) | <0.001 |
| Grade |  | | |  |  |  |
| 1 | Ref | | |  | Ref |  |
| 2 | 3.064(1.346-6.975) | | | **0.008** | 2.880(1.264-6.566) | **0.012** |
| 3 | 3.091(1.285-7.436) | | | **0.012** | 2.761(1.145-6.660) | **0.024** |
| 4 | 10.137(2.846-36.111) | | | **< 0.001** | 6.456(1.758-23.707) | **0.005** |
| Intraoperative blood loss | | |  |  |  |  |
| ≤200ml | Ref | | |  |  |  |
| >200ml | 2.352(1.608-3.440) | | | **< 0.001** | 1.760(1.180-2.625) | **0.006** |
| PA-TACE | |  | |  |  |  |
| No | Ref | | |  | Ref |  |
| Yes | 0.756(0.540-1.058) | | | 0.103 | 0.662(0.470-0.993) | **0.018** |
| **Age>65** |  | | |  |  |  |
| Tumor size | 0.997(0.989-1.005) | | | 0.504 |  |  |
| Grade |  | | |  |  |  |
| 1 | Ref | | |  | Ref |  |
| 2 | 2.050(0.788-5.333) | | | 0.141 | 2.050(0.788-5.333) | 0.141 |
| 3 | 2.906(1.086-7.774) | | | 0.034 | 2.906(1.086-7.774) | 0.034 |
| 4 | - | | | - | - | - |
| Intraoperative blood loss | | |  |  |  |  |
| ≤200ml | Ref | | |  |  |  |
| >200ml | 1.318(0.757-2.292) | | | 0.329 |  |  |
| PA-TACE | |  | |  |  |  |
| No | Ref | | | 0.989 |  |  |
| Yes | 1.004(0.586-1.721) | | |  |  |  |

Supplementary table 4. Trend test and the values of per 1 SD of the whole cohort and the subgroup in validation cohort.

| Tumor size | n | HR (95% CI) | |
| --- | --- | --- | --- |
|  |  | Model 1 | Model 2 |
| **Whole cohort** | 707 |  |  |
| Q 1 (≤22 mm) | 509 | 1.000 | 1.000 |
| Q 2 (23-35 mm) | 483 | 1.199(0.734-1.956) | 1.013(0.672-1.811) |
| Q 3 (36-60 mm) | 464 | 2.091(1.342-3.259) | 2.027(1.294-3.176) |
| Q 4 (≥61 mm) | 464 | 3.323(2.164-5.102) | 3.144(1.990-4.872) |
| P for trend |  | **＜0.001** | **＜0.001** |
| Per 1 SD |  | 1.423(1.276-1.586) | 1.404(1.240-1.589) |
| **Age** ≤65 | 1194 |  |  |
| Q 1 (≤21 mm) | 320 | 1.000 | 1.000 |
| Q 2 (22-31 mm) | 278 | 1.215(0.695-2,122) | 1.124(0.638-1.979) |
| Q 3 (32-49 mm) | 298 | 2.133(1.267-3.589) | 1.996(1.175-3.390) |
| Q 4 (≥50 mm) | 298 | 4.135(2.570-6.711) | 3.874(2.346-6.399) |
| P for trend |  | **＜0.001** | **＜0.001** |
| Per 1 SD |  | 1.632(1.434-1.856) | 1.524(1.329-1.747) |

Model 1: Adjusted for age, sex

Model 2: Adjusted for age, sex, grade, Intraoperative blood loss and PA-TACE
